# Density functional theory calculated data of different electronic states and bond stretch isomers of tris(trifluoroacetylacetonato)-manganese(III)

**DOI:** 10.1016/j.dib.2019.104758

**Published:** 2019-11-06

**Authors:** Jeanet Conradie

**Affiliations:** Department of Chemistry, PO Box 339, University of the Free State, Bloemfontein, 9300, South Africa

**Keywords:** Jahn–Teller, DFT, Elongation, Manganese(III)trifluoroacetylacetonato, High-spin

## Abstract

In this data article, using density functional theory calculations, it is shown that in the gas phase, free from crystal packing effects, different elongation and compression Jahn-Teller geometries of *fac* and *mer* tris(trifluoroacetylacetonato)-manganese(III) are possible. A careful construction of input geometries made it possible to obtain the density functional theory calculated optimized geometries of different elongation and compression Jahn-Teller geometries of *fac* and *mer* tris(trifluoroacetylacetonato)-manganese(III). The *mer* CF_3_–CF_3_ elongation isomer has the lowest energy (Fig. 1), while in the solid state a *mer* CH_3_–CH_3_ compression tris(trifluoroacetylacetonato)-manganese(III) isomer is experimentally characterized [1]. The rare experimental example of a compression tris(β-diketonato)-manganese(III) structure is ascribed to intermolecular F⋯F and F⋯H interactions between the tris(trifluoroacetylacetonato)-manganese(III) molecules in the solid crystalline state, contributing to the distortion of the coordination polyhedron of tris(trifluoroacetylacetonato)-manganese(III) from the expected elongation Jahn-Teller geometry, to the observed higher energy electronic state with compression Jahn-Teller distortion.

**Table:** Specifications Table

Subject	Chemistry
Specific subject area	Computational and structural chemistry.
Type of data	Table Image Figure
How data were acquired	Electronic structure calculations, using the Amsterdam Density Functional (ADF) 2018 programme.
Data format	Raw Analyzed
Parameters for data collection	Suitable xyz coordinates for the input geometries were constructed using CHEMCRAFT. The input coordinates were used in the input file of the ADF program, an example input file is provided in the supplementary information.
Description of data collection	Data were collected from ADF output files
Data source location	Department of Chemistry, University of the Free State, Nelson Mandela Street, Bloemfontein, South Africa
Data accessibility	With the article
Related research article	Roxanne Gostynski, Petrus H.van Rooyen, Jeanet Conradie X-ray diffraction and QTAIM calculations of the non-covalent intermolecular fluorine-fluorine interactions in tris(trifluoroacetylacetonato)-manganese(III). Journal of Molecular Structure 1201 (2020) 127119, https://doi.org/10.1016/j.molstruc.2019.127119

**Table:** 

**Value of the Data** • DFT calculated optimized structural data (coordinates) of different *fac* and *mer* tris(trifluoroacetylacetonato)-manganese(III) isomers are provided for structural and computational chemistry researchers. • Data provide geometrical and electronic structure of elongation and compression Jahn-Teller geometries of *fac* tris(trifluoroacetylacetonato)-manganese(III). • Data provide geometrical and electronic structure of elongation Jahn-Teller geometries of three different bond stretch isomers of *mer* tris(trifluoroacetylacetonato)-manganese(III). • This data can be used to understand the different electron occupation of elongation and compression Jahn-Teller geometries of high spin tris(trifluoroacetylacetonato)-manganese(III). • This data can be used to visualize the molecular orbitals involved in elongation or compression Jahn-Teller geometries of high spin tris(trifluoroacetylacetonato)-manganese(III).

## 1 Data

**Fig. 1:**
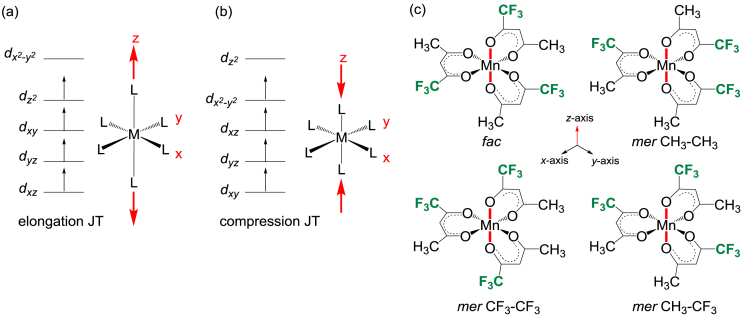
Illustration of the theoretical splitting of the energy levels containing d-electrons of high spin S = 2, d^4^ (t_2g_^3^e_g_^1^) octahedral transition metal complexes from octahedral, due a Jahn-Teller stabilization energy, leading to either (a) a tetragonal elongation (z-out) or (b) a tetragonal compression (z-in) geometry. (c) The z-axis can be aligned along different O–Mn–O bonds for the *fac* (one unique possibility) and *mer* (three different possibilities) isomers of tris(trifluoroacetylacetonato)-manganese(III).

**Fig. 2:**
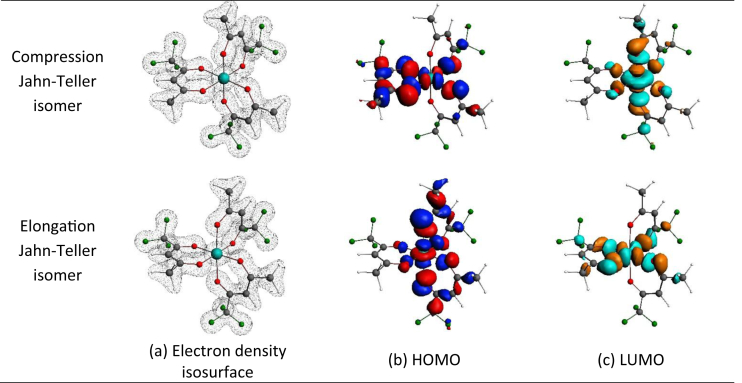
Visualization of the (a) Electron density isosurface (contour 0.08 e/Å^3^), (b) HOMO and (c) LUMO (contour 0.03 e/Å^3^) of the compression (top) and elongation (bottom) of B3LYP-D3/TZP optimized *fac* tris(trifluoroacetylacetonato)-manganese(III). Colour code use for molecule: C (grey), O (red), H (white), F (green) and Mn (turquoise).

**Fig. 3:**
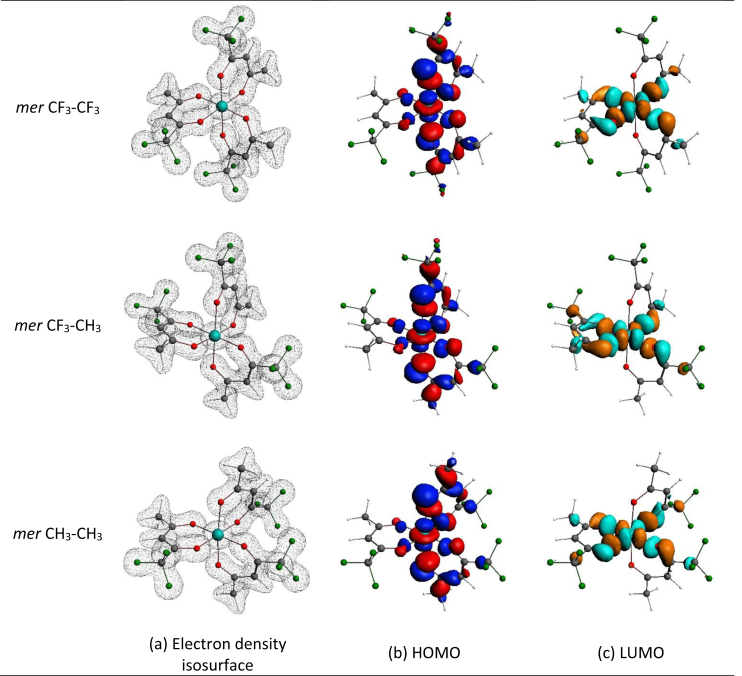
Visualization of the (a) Electron density isosurface (contour 0.08 e/Å^3^), (b) HOMO and (c) LUMO (contour 0.03 e/Å^3^) of the different elongation isomers of B3LYP-D3/TZP optimized *mer* tris(trifluoroacetylacetonato)-manganese(III). Colour code use for molecule: C (grey), O (red), H (white), F (green) and Mn (turquoise).

In Fig. 1 the splitting of the molecular energy levels containing d-electrons for high spin, S = 2, d^4^ (t_2g_^3^e_g_^1^) octahedral transition metal complexes such as tris(trifluoroacetylacetonato)-manganese(III) are illustrated [2,3]. The splitting leads to either elongation (z-out) Jahn-Teller distortion with the highest molecular orbital (HOMO) of $d_{z^{2}}$ character, or to compression (z-in) Jahn-Teller distortion with a HOMO of $d_{x^{2}-y^{2}}$ character. For high spin d^4^ *mer* tris(trifluoroacetylacetonato)-manganese(III) the elongation (or compression) can occur along three different O–Mn–O bonds, leading to three different bond stretch isomers for *mer* tris(trifluoroacetylacetonato)-manganese(III). Although only one *mer* tris(trifluoroacetylacetonato)-manganese(III) isomer is experimentally characterized by solid state crystal data [1], density functional theory calculations can determine the structure of the different electronic state and bond stretch isomers for both *fac* and *mer* tris(trifluoroacetylacetonato)-manganese(III). Fig. 2 shows the electron density isosurface, as well as the highest occupied molecular orbital (HOMO) and lowest unoccupied orbital (LUMO) of both the elongation and compression Jahn-Teller structures of B3LYP-D3/TZP optimized *fac* tris(trifluoroacetylacetonato)-manganese(III). The elongation structure is 0.05 eV lower in energy than the compression *fac* tris(trifluoroacetylacetonato)-manganese(III) structure. The HOMO of the elongation Jahn-Teller structure is of $d_{z^{2}}$ character and the LUMO of $d_{x^{2}-y^{2}}$ character, in agreement with the theoretical splitting of the molecular orbital energy levels as shown in Fig. 1(a). The HOMO of the compression Jahn-Teller structure is of $\;d_{x^{2}-y^{2}}$ character and the LUMO of $\;d_{z^{2}}$ character, in agreement with the theoretical splitting of the molecular orbital energy levels as shown in Fig. 1(b). Fig. 3 shows the electron density isosurface, as well as the HOMO and LUMO of the different elongation Jahn-Teller structures of B3LYP-D3/TZP optimized *mer* tris(trifluoroacetylacetonato)-manganese(III). Table 1 provides the relative energies of the different elongation isomers of tris(trifluoroacetylacetonato)-manganese(III) as obtained by a selection of DFT functionals. The energies of the different isomers are very near to each other, implying that all isomers may exist, though all functionals predict the *mer* CF_3_–CF_3_ isomer to be the most stable.

**Table 1:** Relative energies of the different elongation isomers of *fac* and *mer* tris(trifluoroacetylacetonato)-manganese(III) optimized with the indicated functionals.

Isomer	ΔE (eV)				
	B3LYP-D3	BP86-D3	M06-L	PW91	OLYP^a^
*fac*	0.024	0.046	0.117	0.036	0.044
*mer* CF_3_–CF_3_	0.000	0.000	0.000	0.000	0.000
*mer* CF_3_–CH_3_	0.017	0.029	0.026	0.029	0.030
*mer* CH_3_–CH_3_	0.011	0.023	0.025	0.054	0.064

a From Ref. [4].

## 2 Experimental design, materials, and methods

Density functional theory (DFT) calculations were performed in the gas phase on the neutral molecule, using the Amsterdam Density Functional (ADF) 2018 programme [5]. Results obtained by five different functionals in combination with the TZP (Triple ζ polarized) basis set are reported, namely: OLYP (Handy-Cohen and Lee-Yang-Parr) [[6], [7], [8], [9]], B3LYP-D3 [7,10], PW91 [11], BP86-D3 [12,13] and M06-L [14,15]. Input coordinates were constructed using ChemCraft [16]. Chemcraft and ADF gui was used to visualize the ADF output and t21 files respectively. The optimized coordinates, as well as an example input file, are provided in the supplementary information.

The DFT optimization process are very sensitive to the input geometry, since difference in the energies of the different isomers are small. In some cases the “NumericalQuality good” and “ExactDensity” options in the input file led to the required isomer. An example input file, as well as the optimized coordinates as obtained by the different functionals, and a set of output files, are provided in the supplementary information.

## Appendix A Supplementary data

Multimedia component 1 Multimedia component 1

Multimedia component 2 Input and output files and optimized coordinates of DFT calculations. Multimedia component 2
