# Supplementary material for: Density functional theory calculated data of different electronic states and bond stretch isomers of tris(trifluoroacetylacetonato)-manganese(III)
Source: Data Brief. 2019 Nov 6;27:104758. doi: 10.1016/j.dib.2019.104758 (PMC6864354; doi:10.1016/j.dib.2019.104758)
Supplement: Multimedia component 2 — Input and output files and optimized coordinates of DFT calculations. [file mmc2.doc]

**Article Title**

Density functional theory calculated data of different electronic states and bond stretch isomers of tris(trifluoroacetylacetonato)-manganese(III).

**Authors**

Jeanet Conradie

**Affiliations**

Department of Chemistry, PO Box 339, University of the Free State, Bloemfontein, 9300, South Africa

**Corresponding author(s)**

Jeanet Conradie (conradj@ufs.ac.za)

Supporting information

# Table of Contents

[Table of Contents 1](#__RefHeading___Toc18967299)

[EXAMPLE INPUTFILE 2](#__RefHeading___Toc18967300)

[OPTIMIZED COORDINATES 3](#__RefHeading___Toc18967301)

[1. *fac* elongation (B3LYP-D3) 3](#__RefHeading___Toc18967302)

[2. *fac* compression (B3LYP-D3) 4](#__RefHeading___Toc18967303)

[3. *mer* elongation CF_3_-CF_3_ (B3LYP-D3) 5](#__RefHeading___Toc18967304)

[4. *mer* elongation CF_3_-CH_3_ (B3LYP-D3) 6](#__RefHeading___Toc18967305)

[5. *mer* elongation CH_3_-CH_3_ (B3LYP-D3) 7](#__RefHeading___Toc18967306)

[6. *fac* elongation (BP86-D3) 8](#__RefHeading___Toc18967307)

[7. *mer* elongation CF_3_-CF_3_ (BP86-D3) 9](#__RefHeading___Toc18967308)

[8. *mer* elongation CF_3_-CH_3_ (BP86-D3) 10](#__RefHeading___Toc18967309)

[9. *mer* elongation CH_3_-CH_3_ (BP86-D3) 11](#__RefHeading___Toc18967310)

[10. *fac* elongation (M06-L) 12](#__RefHeading___Toc18967311)

[11. *mer* elongation CF_3_-CF_3_ (M06-L) 13](#__RefHeading___Toc18967312)

[12. *mer* elongation CF_3_-CH_3_ (M06-L) 14](#__RefHeading___Toc18967313)

[13. *mer* elongation CH_3_-CH_3_ (M06-L) 14](#__RefHeading___Toc18967314)

[14. *fac* elongation (PW91) 15](#__RefHeading___Toc18967315)

[15. *mer* elongation CF_3_-CF_3_ (PW91) 16](#__RefHeading___Toc18967316)

[16. *mer* elongation CF_3_-CH_3_ (PW91) 17](#__RefHeading___Toc18967317)

[17. *mer* elongation CH_3_-CH_3_ (PW91) 18](#__RefHeading___Toc18967318)

# EXAMPLE INPUTFILE

Title Mntfaa3

COMMENT

ADF2018 DEFAULT integration, geometry and scf

END

UNITS

length angstrom

END

Atoms Cartesian

Mn 0.000000000 0.000000000 0.051100000

O 1.838400000 0.632500000 0.080600000

O 0.524700000 -1.444000000 1.512800000

O -1.838400000 -0.632500000 0.080600000

O -0.524700000 1.444000000 1.512800000

O -0.418000000 1.387300000 -1.494100000

O 0.418000000 -1.387300000 -1.494100000

C 4.131100000 0.936100000 0.481200000

C 2.845300000 0.181500000 0.747100000

C 2.835900000 -0.886800000 1.646100000

C 1.684900000 -1.637900000 1.985800000

C 1.808800000 -2.766000000 2.993400000

C -4.131100000 -0.936100000 0.481200000

C -2.845300000 -0.181500000 0.747100000

C -2.835900000 0.886800000 1.646100000

C -1.684900000 1.637900000 1.985800000

C -1.808800000 2.766000000 2.993400000

C -0.705300000 2.414800000 -3.602000000

C -0.355800000 1.202400000 -2.754100000

C 0.000000000 0.000000000 -3.398100000

C 0.355800000 -1.202400000 -2.754100000

C 0.705300000 -2.414800000 -3.602000000

H 3.986600000 1.993400000 0.727300000

H 4.368300000 0.881700000 -0.586500000

H 4.968600000 0.541400000 1.058900000

H 3.773800000 -1.146600000 2.125000000

H -3.773800000 1.146600000 2.125000000

H -1.490900000 3.701300000 2.520600000

H -1.125500000 2.577800000 3.828200000

H -2.824300000 2.883000000 3.377100000

H -1.718300000 2.749000000 -3.352900000

H -0.646400000 2.214600000 -4.674000000

H -0.025200000 3.236100000 -3.351300000

H 0.000000000 0.000000000 -4.483600000

F -0.151500000 -3.449500000 -3.286200000

F 0.630900000 -2.161800000 -4.956600000

F 1.981500000 -2.835800000 -3.288200000

F -4.430000000 -0.867600000 -0.864300000

F -3.949100000 -2.268400000 0.791300000

F -5.190200000 -0.437000000 1.211800000

F 0.947700000 -2.528900000 4.045400000

F 1.408200000 -3.944500000 2.397700000

F 3.092300000 -2.913900000 3.478300000

end

SYMMETRY nosym

CHARGE 0 4

unrestricted

NumericalQuality good

ExactDensity

GEOMETRY

Iterations 200

END

SCF

Iterations 200

END

BASIS

Type TZP

Core none

END

XC

hybrid b3lyp

DISPERSION Grimme3

END

endinput

# OPTIMIZED COORDINATES

# *fac* elongation (B3LYP-D3)

Mn 0.283841000 0.081275000 0.114915000

O 2.147088000 0.662860000 0.216339000

O 0.607175000 -1.253398000 1.507778000

O -1.548015000 -0.528976000 -0.009866000

O -0.357252000 1.485844000 1.632036000

O -0.045029000 1.350831000 -1.314208000

O 0.823312000 -1.374461000 -1.395612000

C 3.058748000 0.113648000 0.911575000

C 2.943177000 -0.941112000 1.798364000

C 1.709772000 -1.570773000 2.058258000

C -2.606094000 -0.126752000 0.596848000

C -2.665412000 0.880645000 1.562615000

C -1.545133000 1.593002000 2.012010000

C -0.177776000 1.084484000 -2.561653000

C 0.071223000 -0.085051000 -3.239089000

C 0.582449000 -1.269749000 -2.622083000

H 3.823175000 -1.282282000 2.318232000

H -3.625741000 1.113644000 1.993029000

H -0.104273000 -0.098275000 -4.302757000

C -1.747561000 2.621377000 3.158406000

F -1.128968000 3.784668000 2.881836000

F -3.053703000 2.895818000 3.402694000

F -1.217135000 2.127756000 4.306223000

C 4.450973000 0.737722000 0.661494000

F 4.405606000 2.077077000 0.777938000

F 5.387704000 0.276657000 1.524387000

F 4.865060000 0.434762000 -0.591819000

C 1.629755000 -2.704084000 3.041281000

H 0.884816000 -2.461261000 3.800746000

H 1.278117000 -3.593968000 2.515153000

H 2.586187000 -2.913328000 3.514736000

C -3.856240000 -0.858098000 0.185265000

H -3.997847000 -0.733069000 -0.889828000

H -3.716917000 -1.924659000 0.371959000

H -4.738830000 -0.505581000 0.714177000

C -0.704593000 2.307332000 -3.345980000

F 0.116168000 3.366582000 -3.187929000

F -0.820176000 2.072264000 -4.675679000

F -1.930986000 2.659255000 -2.889461000

C 0.874662000 -2.473320000 -3.483919000

H 1.948273000 -2.671760000 -3.448918000

H 0.371177000 -3.341598000 -3.056061000

H 0.567072000 -2.340516000 -4.519305000

# *fac* compression (B3LYP-D3)

Mn -0.036310000 0.008140000 0.111908000

O 1.762238000 0.645976000 0.100860000

O 0.470500000 -1.362791000 1.578053000

O -1.836581000 -0.627672000 0.102547000

O -0.546383000 1.402491000 1.549326000

O -0.512962000 1.340146000 -1.396182000

O 0.468931000 -1.349977000 -1.359573000

C 4.091561000 0.804839000 0.248137000

C 2.808093000 0.125483000 0.631620000

C 2.819056000 -0.967511000 1.501932000

C 1.652991000 -1.603988000 1.926436000

C 1.763212000 -2.726326000 2.986080000

C -4.147863000 -0.844368000 0.359069000

C -2.835338000 -0.123232000 0.723988000

C -2.865340000 0.922704000 1.614112000

C -1.697285000 1.634196000 2.004136000

C -1.806116000 2.734408000 3.025023000

C -0.844197000 2.357407000 -3.490608000

C -0.416285000 1.182669000 -2.648664000

C 0.051566000 0.010219000 -3.285032000

C 0.448509000 -1.131020000 -2.604719000

C 0.969098000 -2.334852000 -3.424509000

H 4.004204000 1.872941000 0.453092000

H 4.235683000 0.690771000 -0.828497000

H 4.950032000 0.394549000 0.774871000

H 3.765225000 -1.307092000 1.889714000

H -3.811981000 1.199133000 2.048570000

H -1.424103000 3.657301000 2.584739000

H -1.161896000 2.488846000 3.871425000

H -2.825148000 2.887616000 3.373627000

H -1.896044000 2.567357000 -3.285897000

H -0.710179000 2.184378000 -4.556524000

H -0.273474000 3.236167000 -3.184971000

H 0.097095000 -0.000377000 -4.361880000

F 0.267608000 -3.448243000 -3.135895000

F 0.899394000 -2.142872000 -4.763101000

F 2.268003000 -2.571361000 -3.119302000

F -4.410623000 -0.682461000 -0.956626000

F -4.051593000 -2.163337000 0.607407000

F -5.210652000 -0.370877000 1.048308000

F 1.177526000 -2.323857000 4.137055000

F 1.138462000 -3.839991000 2.565223000

F 3.044062000 -3.054628000 3.274324000

# *mer* elongation CF_3_-CF_3_ (B3LYP-D3)

Mn 0.163144000 -0.032881000 0.057619000

O 1.985933000 0.602813000 0.191034000

O 0.649561000 -1.533613000 1.529340000

O -1.676162000 -0.672626000 -0.090886000

O -0.363674000 1.209122000 1.473905000

O -0.355004000 1.483418000 -1.419277000

O 0.638748000 -1.200793000 -1.406224000

C 4.252340000 0.931439000 0.673353000

C 2.972363000 0.169576000 0.884845000

C 2.937505000 -0.905891000 1.777843000

C 1.787795000 -1.660478000 2.034597000

C 1.876676000 -2.800720000 3.080942000

C -4.010285000 -0.787465000 0.009497000

C -2.688532000 -0.127004000 0.451490000

C -2.717797000 0.920352000 1.349807000

C -1.540239000 1.525533000 1.836092000

C -1.621676000 2.622142000 2.858253000

C -1.173370000 2.341498000 -3.446053000

C -0.562505000 1.183276000 -2.616625000

C -0.304392000 -0.030712000 -3.263098000

C 0.287706000 -1.132406000 -2.638981000

C 0.573788000 -2.380566000 -3.426754000

H 4.076574000 1.985407000 0.895907000

H 4.529293000 0.866372000 -0.380729000

H 5.066626000 0.555898000 1.289152000

H 3.844690000 -1.159915000 2.301259000

H -3.670548000 1.264653000 1.716516000

H -1.189421000 3.527478000 2.426904000

H -1.009485000 2.348147000 3.719000000

H -2.642222000 2.820293000 3.177313000

F -2.332968000 2.751914000 -2.887056000

F -1.438631000 2.003215000 -4.729774000

F -0.328452000 3.395368000 -3.470400000

H -0.550653000 -0.114665000 -4.308911000

H -0.008681000 -3.199939000 -3.000095000

H 0.331858000 -2.271485000 -4.481646000

H 1.627677000 -2.639747000 -3.312981000

F -4.125073000 -0.744478000 -1.333513000

F -4.035005000 -2.080435000 0.391336000

F -5.096826000 -0.180495000 0.535894000

F 1.057323000 -2.534443000 4.123374000

F 1.490633000 -3.972362000 2.536931000

F 3.124061000 -2.973894000 3.580400000

# *mer* elongation CF_3_-CH_3_ (B3LYP-D3)

Mn 1.925022000 -0.371620000 16.712883000

O 2.651080000 1.059847000 17.829939000

O 3.425486000 -0.182022000 15.477349000

O 0.863281000 1.177460000 15.613555000

O 0.412359000 -0.491535000 17.905087000

O 1.192145000 -1.773909000 15.594704000

O 2.950073000 -1.909309000 17.829506000

F 2.596312000 3.697063000 18.650972000

F 4.118457000 2.518513000 19.681908000

F 4.696447000 3.904597000 18.099997000

F -1.756084000 3.528768000 15.717980000

F -0.854326000 2.595657000 13.965743000

F 0.340016000 3.904741000 15.243004000

F 0.294008000 -5.128046000 15.027310000

F -0.956545000 -3.348843000 14.868835000

F 0.770130000 -3.592849000 13.554206000

C 3.553848000 1.878649000 17.465932000

C 4.334587000 1.839870000 16.328777000

H 5.062879000 2.617635000 16.168974000

C 4.247315000 0.783864000 15.396984000

C -0.156626000 1.734637000 16.077043000

C -0.886622000 1.394631000 17.222061000

H -1.751153000 1.988364000 17.469998000

C -0.578707000 0.312231000 18.049310000

C 1.195430000 -3.033996000 15.826163000

C 1.854314000 -3.741935000 16.799885000

H 1.727512000 -4.812588000 16.823473000

C 2.725264000 -3.140078000 17.761239000

C 3.750357000 3.019593000 18.484456000

C -0.624459000 2.955788000 15.244656000

C 0.318430000 -3.792148000 14.810705000

C 5.163921000 0.748672000 14.208880000

H 5.956786000 1.490554000 14.271716000

H 5.588006000 -0.251194000 14.112884000

H 4.564718000 0.934590000 13.313986000

C -1.449236000 -0.000552000 19.234853000

H -2.305413000 0.666147000 19.309313000

H -1.791965000 -1.034013000 19.159594000

H -0.844532000 0.076588000 20.140865000

C 3.401238000 -4.033712000 18.770098000

H 3.603018000 -5.026780000 18.369408000

H 4.321278000 -3.565710000 19.114736000

H 2.733841000 -4.146306000 19.629720000

# *mer* elongation CH_3_-CH_3_ (B3LYP-D3)

Mn 0.410793000 -0.101876000 0.198902000

O 2.257541000 0.545937000 0.248259000

O 0.865317000 -1.509601000 1.468778000

O -1.421896000 -0.737900000 0.147254000

O -0.205329000 1.231264000 1.792022000

O -0.041842000 1.271383000 -1.074305000

O 0.860129000 -1.401474000 -1.483245000

C 3.216119000 0.021923000 0.894263000

C 3.184139000 -1.077143000 1.731969000

C 1.994197000 -1.788164000 1.983350000

C -2.450966000 -0.058563000 0.509447000

C -2.520367000 1.049725000 1.310532000

C -1.378855000 1.639990000 1.944700000

C -0.382296000 1.081650000 -2.300176000

C -0.223891000 -0.027835000 -3.084683000

C 0.422261000 -1.229096000 -2.642294000

H 4.094758000 -1.394509000 2.212447000

H -3.489808000 1.483459000 1.498070000

H -0.579677000 0.013010000 -4.102141000

C 4.551339000 0.760833000 0.664597000

F 4.445977000 2.048224000 1.047405000

F 5.576012000 0.208634000 1.354049000

F 4.877177000 0.735487000 -0.643799000

C 1.997431000 -2.965152000 2.916151000

H 1.289748000 -2.772938000 3.724651000

H 1.637038000 -3.841027000 2.373480000

H 2.982985000 -3.166757000 3.329491000

C -1.606342000 2.831530000 2.838065000

H -2.509500000 2.718546000 3.438556000

H -0.738956000 2.986009000 3.475718000

H -1.737280000 3.714557000 2.205481000

C -3.746449000 -0.648861000 -0.075921000

F -3.684712000 -0.665929000 -1.426580000

F -3.919532000 -1.922181000 0.343554000

F -4.851819000 0.047568000 0.272010000

C 0.573612000 -2.348426000 -3.639744000

H 0.987772000 -1.979873000 -4.579828000

H 1.208305000 -3.128890000 -3.227418000

H -0.413831000 -2.764269000 -3.858584000

C -1.055175000 2.334286000 -2.888897000

F -0.225446000 3.397033000 -2.822284000

F -1.419755000 2.181353000 -4.181821000

F -2.170144000 2.637106000 -2.184912000

# *fac* elongation (BP86-D3)

Mn 0.060434000 -0.313448000 0.089513000

O 1.895070000 0.365988000 -0.074785000

O 0.609427000 -1.656978000 1.391877000

O -1.774707000 -0.900975000 0.273179000

O -0.261190000 1.078277000 1.722885000

O -0.511832000 1.048253000 -1.160254000

O 0.467855000 -1.626546000 -1.569434000

C 2.820759000 0.109878000 0.772938000

C 2.791990000 -0.786513000 1.834874000

C 1.698167000 -1.651021000 2.072863000

C -2.763310000 -0.196401000 0.724516000

C -2.651539000 0.964287000 1.507103000

C -1.430644000 1.499003000 1.956043000

C -0.158117000 1.100435000 -2.404442000

C 0.433990000 0.129585000 -3.186002000

C 0.722273000 -1.201702000 -2.734106000

H 3.670750000 -0.870398000 2.468026000

H -3.569642000 1.457748000 1.813885000

H 0.668775000 0.382706000 -4.217135000

C -1.469050000 2.758683000 2.868092000

F -0.865380000 3.808173000 2.244604000

F -2.737374000 3.149988000 3.200655000

F -0.798227000 2.520772000 4.031631000

C 4.129626000 0.881431000 0.472017000

F 3.880865000 2.188956000 0.222603000

F 5.026426000 0.811867000 1.498239000

F 4.727098000 0.343282000 -0.634730000

C 1.758436000 -2.659387000 3.189136000

H 1.119569000 -2.300476000 4.011044000

H 1.339975000 -3.612830000 2.842585000

H 2.775792000 -2.804502000 3.568314000

C -4.121749000 -0.726036000 0.342498000

H -4.255171000 -0.594726000 -0.741857000

H -4.162794000 -1.804596000 0.546292000

H -4.933328000 -0.213379000 0.870433000

C -0.497560000 2.473639000 -3.028230000

F 0.140116000 3.474636000 -2.364221000

F -0.144594000 2.559648000 -4.344951000

F -1.840654000 2.707326000 -2.947810000

C 1.379440000 -2.150967000 -3.708618000

H 2.432646000 -1.854587000 -3.837728000

H 1.342454000 -3.172759000 -3.316510000

H 0.904039000 -2.102964000 -4.697974000

# *mer* elongation CF_3_-CF_3_ (BP86-D3)

Mn -0.043155000 -0.239304000 0.249957000

O 1.771313000 0.458331000 0.021550000

O 0.547692000 -1.565347000 1.551203000

O -1.858244000 -0.873987000 0.460043000

O -0.393520000 1.207287000 1.810935000

O -0.641111000 1.045917000 -1.065795000

O 0.399550000 -1.621126000 -1.374788000

C 2.812986000 -0.035456000 0.583387000

C 2.889072000 -1.086450000 1.487147000

C 1.749970000 -1.793770000 1.937350000

C -2.862701000 -0.211618000 0.936034000

C -2.781559000 0.975455000 1.683279000

C -1.573314000 1.577375000 2.075502000

C -0.208558000 1.150211000 -2.284343000

C 0.470568000 0.150978000 -2.997285000

C 0.710799000 -1.144707000 -2.503573000

H 3.864667000 -1.371239000 1.870138000

H -3.711192000 1.428275000 2.017320000

H 0.800427000 0.382767000 -4.006334000

C -1.637526000 2.852817000 2.964329000

F -1.055518000 3.903803000 2.319885000

F -2.913096000 3.225748000 3.288947000

F -0.961577000 2.650830000 4.129800000

C 1.416085000 -2.163804000 -3.444706000

F 1.924074000 -1.592869000 -4.578588000

F 2.446202000 -2.775893000 -2.799006000

F 0.532312000 -3.129278000 -3.834283000

C 4.108753000 0.689932000 0.149128000

F 4.059750000 2.000251000 0.513021000

F 5.227657000 0.144103000 0.706228000

F 4.247903000 0.631619000 -1.204052000

C 1.895404000 -2.899573000 2.947896000

H 1.364623000 -2.608246000 3.866083000

H 1.404629000 -3.803723000 2.562756000

H 2.942764000 -3.113944000 3.185027000

C -4.204537000 -0.828739000 0.635102000

H -4.380788000 -0.777420000 -0.449707000

H -4.182472000 -1.892876000 0.907194000

H -5.023140000 -0.325352000 1.160835000

C -0.490673000 2.484438000 -2.923173000

H 0.108114000 3.250736000 -2.408528000

H -0.251762000 2.493853000 -3.992242000

H -1.547255000 2.744172000 -2.772649000

# *mer* elongation CF_3_-CH_3_ (BP86-D3)

Mn 0.092303000 -0.184626000 0.335573000

O 1.908330000 0.562152000 0.175199000

O 0.690204000 -1.544814000 1.582469000

O -1.717428000 -0.908746000 0.449715000

O -0.414202000 1.181278000 1.918932000

O -0.503819000 1.129214000 -0.938312000

O 0.574889000 -1.551270000 -1.281856000

C 2.958196000 -0.002812000 0.643813000

C 3.044065000 -1.154041000 1.418163000

C 1.903529000 -1.863119000 1.859645000

C -2.736064000 -0.238423000 0.873679000

C -2.767241000 0.925200000 1.620109000

C -1.598152000 1.583923000 2.123048000

C -0.346523000 1.133025000 -2.225266000

C 0.165735000 0.082223000 -2.998832000

C 0.568851000 -1.162918000 -2.483227000

H 4.025920000 -1.503230000 1.724430000

H -3.739795000 1.343946000 1.867312000

H 0.236509000 0.235440000 -4.072080000

C 1.061761000 -2.244068000 -3.487514000

F 1.145594000 -1.793277000 -4.776239000

F 2.298379000 -2.691384000 -3.129319000

F 0.210451000 -3.308397000 -3.480811000

C 4.252894000 0.760748000 0.272075000

F 4.209671000 2.030380000 0.762581000

F 5.376191000 0.163804000 0.766018000

F 4.382926000 0.835950000 -1.081224000

C 2.055267000 -3.087792000 2.720314000

H 1.373805000 -3.015589000 3.578338000

H 1.741899000 -3.962539000 2.130869000

H 3.084464000 -3.233628000 3.064847000

C -0.780676000 2.416700000 -2.884006000

H -0.180147000 3.243982000 -2.479137000

H -0.671275000 2.383283000 -3.973310000

H -1.829454000 2.617424000 -2.623641000

C -1.786982000 2.808120000 2.989605000

H -2.615175000 3.433602000 2.630453000

H -2.034765000 2.487143000 4.013784000

H -0.856879000 3.385662000 3.021046000

C -4.066942000 -0.894079000 0.438880000

F -4.170799000 -0.888709000 -0.923030000

F -4.124658000 -2.188653000 0.856008000

F -5.166449000 -0.251930000 0.935669000

# *mer* elongation CH_3_-CH_3_ (BP86-D3)

Mn 0.440636000 -0.109905000 0.279267000

O 2.260677000 0.615378000 0.360632000

O 0.891516000 -1.541218000 1.508489000

O -1.370059000 -0.831623000 0.149342000

O -0.255053000 1.145329000 1.881157000

O -0.047632000 1.302730000 -0.942775000

O 0.977073000 -1.338768000 -1.422003000

C 3.239906000 0.058967000 0.968609000

C 3.222533000 -1.080930000 1.765606000

C 2.040260000 -1.813168000 2.015102000

C -2.423683000 -0.126695000 0.414834000

C -2.532021000 1.016504000 1.179935000

C -1.432282000 1.614891000 1.881450000

C -0.441175000 1.100941000 -2.161442000

C -0.280766000 -0.012364000 -2.957236000

C 0.433687000 -1.191685000 -2.554997000

H 4.150073000 -1.410738000 2.224652000

H -3.510141000 1.484168000 1.261279000

H -0.703011000 0.015583000 -3.958793000

C 4.575626000 0.811419000 0.745383000

F 4.490542000 2.081301000 1.226787000

F 5.630126000 0.202330000 1.363705000

F 4.859274000 0.879950000 -0.583369000

C 2.057099000 -2.995495000 2.946295000

H 1.511632000 -2.723617000 3.862640000

H 1.515720000 -3.830481000 2.482630000

H 3.074372000 -3.302571000 3.211983000

C -1.686510000 2.890381000 2.647425000

H -1.677384000 3.727612000 1.930489000

H -2.668080000 2.886005000 3.139537000

H -0.888244000 3.053122000 3.379274000

C -3.682516000 -0.711285000 -0.263065000

F -3.509783000 -0.763017000 -1.617201000

F -3.916227000 -1.983498000 0.171147000

F -4.809029000 0.019037000 -0.021180000

C 0.527925000 -2.331193000 -3.541842000

H 0.695441000 -1.970849000 -4.565348000

H 1.324407000 -3.020325000 -3.241733000

H -0.429954000 -2.875955000 -3.535463000

C -1.203646000 2.326553000 -2.710012000

F -0.457500000 3.458070000 -2.584418000

F -1.545916000 2.198656000 -4.024013000

F -2.358099000 2.512649000 -2.000926000

# *fac* elongation (M06-L)

Mn 0.191132000 -0.331649000 0.083476000

O 2.045614000 0.336314000 0.011243000

O 0.704668000 -1.681207000 1.388535000

O -1.673490000 -0.896916000 0.216878000

O -0.190390000 1.022745000 1.713373000

O -0.356101000 1.071319000 -1.132156000

O 0.629037000 -1.562809000 -1.611701000

C 2.902066000 0.106632000 0.915665000

C 2.808301000 -0.764140000 1.983010000

C 1.725232000 -1.641934000 2.145730000

C -2.654997000 -0.177665000 0.614805000

C -2.550404000 0.976678000 1.392881000

C -1.345730000 1.468787000 1.897318000

C -0.202712000 1.087109000 -2.402345000

C 0.295495000 0.121770000 -3.235847000

C 0.693151000 -1.174455000 -2.800115000

H 3.635774000 -0.827097000 2.669775000

H -3.459505000 1.487371000 1.664050000

H 0.355821000 0.349917000 -4.287749000

C -1.384400000 2.706364000 2.808837000

F -0.801824000 3.748611000 2.194471000

F -2.632340000 3.078036000 3.151280000

F -0.708109000 2.468976000 3.944486000

C 4.208092000 0.877312000 0.693575000

F 3.964677000 2.168907000 0.457541000

F 5.041943000 0.795279000 1.743528000

F 4.847263000 0.369900000 -0.377054000

C 1.725003000 -2.618176000 3.275755000

H 1.139615000 -2.193771000 4.091932000

H 1.237958000 -3.540070000 2.974380000

H 2.725420000 -2.818266000 3.645609000

C -4.001674000 -0.670436000 0.187875000

H -4.100034000 -0.533863000 -0.888701000

H -4.081640000 -1.737597000 0.380707000

H -4.809839000 -0.143481000 0.684661000

C -0.716271000 2.401840000 -2.994606000

F -0.125561000 3.451426000 -2.410737000

F -0.512285000 2.495549000 -4.319023000

F -2.042830000 2.505631000 -2.781069000

C 1.218970000 -2.123526000 -3.833275000

H 2.223867000 -1.810290000 -4.118666000

H 1.267181000 -3.131208000 -3.435381000

H 0.612967000 -2.104546000 -4.735894000

# *mer* elongation CF_3_-CF_3_ (M06-L)

Mn -0.088068000 -0.103874000 0.375231000

O 1.731958000 0.548037000 0.128136000

O 0.538396000 -1.395672000 1.727266000

O -1.882305000 -0.792942000 0.556913000

O -0.546279000 1.355326000 1.837577000

O -0.695542000 1.116012000 -1.033070000

O 0.416048000 -1.494087000 -1.200134000

C 2.754278000 -0.167990000 0.380174000

C 2.838344000 -1.287644000 1.175283000

C 1.728158000 -1.805272000 1.870557000

C -2.914535000 -0.173247000 0.989016000

C -2.901506000 1.029688000 1.695763000

C -1.729834000 1.675790000 2.090453000

C -0.186561000 1.212773000 -2.203878000

C 0.609202000 0.245213000 -2.817994000

C 0.857810000 -1.013118000 -2.270636000

H 3.802178000 -1.747029000 1.315624000

H -3.845346000 1.444647000 2.008297000

H 1.041212000 0.479135000 -3.776796000

C -1.836422000 2.927201000 2.975088000

F -1.291511000 3.984230000 2.351712000

F -3.104175000 3.249535000 3.292121000

F -1.166760000 2.735521000 4.122540000

C 1.784079000 -1.969014000 -3.036068000

F 2.395134000 -1.390598000 -4.084776000

F 2.742396000 -2.427549000 -2.209180000

F 1.100888000 -3.029679000 -3.498471000

C 4.011672000 0.361339000 -0.316742000

F 4.315569000 1.590618000 0.122972000

F 5.080030000 -0.424718000 -0.117330000

F 3.796904000 0.438790000 -1.640411000

C 1.916458000 -2.908009000 2.858653000

H 1.458563000 -2.624978000 3.803531000

H 1.387116000 -3.791400000 2.505968000

H 2.962012000 -3.151272000 3.012884000

C -4.209512000 -0.863034000 0.701746000

H -4.332882000 -0.955951000 -0.375947000

H -4.175427000 -1.874861000 1.100976000

H -5.059977000 -0.333378000 1.118139000

C -0.510564000 2.484040000 -2.919561000

H -0.114719000 3.324911000 -2.352540000

H -0.111944000 2.505260000 -3.928547000

H -1.590924000 2.611431000 -2.953515000

# *mer* elongation CF_3_-CH_3_ (M06-L)

Mn -0.061236000 -0.110411000 0.510985000

O 1.737525000 0.578801000 0.246050000

O 0.640967000 -1.451745000 1.783733000

O -1.825460000 -0.869272000 0.697449000

O -0.617923000 1.363916000 1.931934000

O -0.710887000 1.103892000 -0.894443000

O 0.426452000 -1.496230000 -1.077322000

C 2.757496000 -0.182326000 0.335360000

C 2.864024000 -1.395749000 0.970457000

C 1.805338000 -1.946011000 1.721780000

C -2.860077000 -0.210639000 1.056471000

C -2.939879000 1.003871000 1.687839000

C -1.800506000 1.735058000 2.123474000

C -0.263699000 1.184490000 -2.090097000

C 0.506899000 0.214477000 -2.733117000

C 0.794442000 -1.032687000 -2.182467000

H 3.810997000 -1.908261000 0.953530000

H -3.920192000 1.395171000 1.904348000

H 0.877142000 0.432315000 -3.721060000

C 1.681976000 -1.997699000 -2.980817000

F 2.064330000 -1.516132000 -4.176119000

F 2.804108000 -2.258941000 -2.275637000

F 1.052706000 -3.162262000 -3.189509000

C 3.978156000 0.412971000 -0.366138000

F 4.341571000 1.561957000 0.225234000

F 5.033716000 -0.413894000 -0.351282000

F 3.684160000 0.685473000 -1.647133000

C 2.036177000 -3.192777000 2.508198000

H 1.457740000 -3.172150000 3.426243000

H 1.674459000 -4.035173000 1.918111000

H 3.088069000 -3.353663000 2.721359000

C -0.636284000 2.439627000 -2.810946000

H -0.257200000 3.297738000 -2.259342000

H -0.256079000 2.461784000 -3.827057000

H -1.720910000 2.530723000 -2.829578000

C -2.019068000 2.996679000 2.900932000

H -2.994294000 3.433574000 2.706802000

H -1.959927000 2.766397000 3.964773000

H -1.233614000 3.713319000 2.681743000

C -4.146277000 -0.953186000 0.687425000

F -4.253569000 -1.049834000 -0.648907000

F -4.135892000 -2.196480000 1.188450000

F -5.251070000 -0.337552000 1.143064000

# *mer* elongation CH_3_-CH_3_ (M06-L)

Mn 0.572341000 -0.281232000 0.022273000

O 2.405591000 0.420426000 0.149701000

O 1.020364000 -1.726897000 1.245415000

O -1.299535000 -0.889883000 -0.023626000

O -0.003036000 0.933696000 1.710385000

O 0.077979000 1.187452000 -1.135460000

O 0.903301000 -1.445846000 -1.731373000

C 3.205201000 0.123561000 1.086062000

C 3.072019000 -0.855616000 2.048833000

C 1.993877000 -1.753693000 2.061444000

C -2.274137000 -0.117961000 0.280317000

C -2.281545000 0.990400000 1.088046000

C -1.138232000 1.460098000 1.793892000

C -0.473226000 1.019768000 -2.282563000

C -0.458640000 -0.079286000 -3.096373000

C 0.262033000 -1.270873000 -2.793207000

H 3.855769000 -0.978390000 2.777134000

H -3.212492000 1.521588000 1.202504000

H -1.000187000 -0.026029000 -4.026663000

C 4.454947000 1.008779000 1.087511000

F 4.115240000 2.268598000 1.402603000

F 5.375944000 0.598479000 1.976719000

F 5.024690000 1.020729000 -0.123043000

C 1.939953000 -2.827104000 3.097924000

H 1.367904000 -2.452767000 3.947437000

H 1.417686000 -3.697364000 2.714156000

H 2.928267000 -3.099217000 3.455327000

C -1.303351000 2.621562000 2.724764000

H -2.168913000 3.228143000 2.475056000

H -1.436974000 2.245411000 3.739101000

H -0.402748000 3.227650000 2.719218000

C -3.574043000 -0.567613000 -0.390372000

F -3.413662000 -0.622853000 -1.726056000

F -3.916286000 -1.798994000 0.025220000

F -4.608320000 0.250092000 -0.138322000

C 0.239765000 -2.384245000 -3.792934000

H 0.124421000 -2.024587000 -4.811369000

H 1.134960000 -2.990948000 -3.703851000

H -0.616824000 -3.022169000 -3.568779000

C -1.261228000 2.259877000 -2.707211000

F -0.516731000 3.366386000 -2.586933000

F -1.703676000 2.191789000 -3.972513000

F -2.338654000 2.405280000 -1.908558000

# *fac* elongation (PW91)

Mn 0.224117000 -0.024676000 0.156564000

O 2.083201000 0.618855000 0.179151000

O 0.628759000 -1.385283000 1.496533000

O -1.609059000 -0.664534000 0.139705000

O -0.329496000 1.391264000 1.709387000

O -0.214314000 1.304619000 -1.185236000

O 0.759910000 -1.406553000 -1.434083000

C 3.017610000 0.141115000 0.909956000

C 2.940538000 -0.894526000 1.832716000

C 1.747832000 -1.608125000 2.081297000

C -2.664599000 -0.120790000 0.650997000

C -2.684902000 0.991790000 1.503747000

C -1.536193000 1.654549000 1.968730000

C -0.101809000 1.189780000 -2.465797000

C 0.335558000 0.117886000 -3.216260000

C 0.756282000 -1.136627000 -2.668280000

H 3.834869000 -1.170382000 2.381218000

H -3.653100000 1.346143000 1.844249000

H 0.360086000 0.236681000 -4.295310000

C -1.720102000 2.857634000 2.936851000

F -1.179045000 3.984081000 2.398960000

F -3.030594000 3.125827000 3.223963000

F -1.086169000 2.612329000 4.119667000

C 4.381508000 0.830338000 0.662130000

F 4.270078000 2.177474000 0.765772000

F 5.339986000 0.419991000 1.542596000

F 4.824151000 0.535756000 -0.595242000

C 1.728079000 -2.719246000 3.095739000

H 1.041581000 -2.446131000 3.909070000

H 1.326554000 -3.628252000 2.628648000

H 2.719478000 -2.920366000 3.509906000

C -3.952914000 -0.794213000 0.259322000

H -4.092305000 -0.700706000 -0.826482000

H -3.884326000 -1.866483000 0.483237000

H -4.817299000 -0.363677000 0.773767000

C -0.574802000 2.472255000 -3.189541000

F 0.030679000 3.573005000 -2.674565000

F -0.322551000 2.446544000 -4.530365000

F -1.925041000 2.625783000 -3.031332000

C 1.253287000 -2.201817000 -3.617335000

H 2.346549000 -2.111180000 -3.702120000

H 1.031987000 -3.192430000 -3.207899000

H 0.827711000 -2.097157000 -4.620855000

# *mer* elongation CF_3_-CF_3_ (PW91)

Mn 0.136824000 -0.142406000 0.172525000

O 1.984241000 0.532456000 0.114011000

O 0.627321000 -1.530910000 1.445146000

O -1.713345000 -0.737999000 0.277346000

O -0.289784000 1.258307000 1.793366000

O -0.341876000 1.180536000 -1.145185000

O 0.562594000 -1.551375000 -1.448700000

C 2.953870000 0.074958000 0.812292000

C 2.942328000 -0.999075000 1.691946000

C 1.782258000 -1.762668000 1.951747000

C -2.721671000 -0.174205000 0.855450000

C -2.662395000 0.926834000 1.723130000

C -1.470939000 1.550213000 2.128705000

C -0.184622000 1.159814000 -2.429270000

C 0.250273000 0.066754000 -3.189848000

C 0.574835000 -1.192489000 -2.657483000

H 3.865120000 -1.271313000 2.193022000

H -3.599189000 1.299435000 2.127280000

H 0.327179000 0.202105000 -4.264009000

C -1.564170000 2.745206000 3.121446000

F -1.015164000 3.861803000 2.567179000

F -2.848049000 3.053864000 3.479182000

F -0.878226000 2.467077000 4.265932000

C 0.990570000 -2.320201000 -3.646339000

F 1.077343000 -1.898261000 -4.943747000

F 2.203806000 -2.832009000 -3.300350000

F 0.080727000 -3.335235000 -3.608615000

C 4.266254000 0.874403000 0.604458000

F 4.128985000 2.133572000 1.103230000

F 5.333334000 0.295048000 1.226760000

F 4.561940000 0.974771000 -0.718267000

C 1.846121000 -2.948812000 2.871597000

H 1.100685000 -2.829554000 3.668473000

H 1.569659000 -3.848465000 2.306152000

H 2.838938000 -3.082604000 3.308938000

C -4.051322000 -0.801598000 0.529672000

H -4.258649000 -0.669861000 -0.541656000

H -3.998766000 -1.881944000 0.714095000

H -4.867686000 -0.365503000 1.111985000

C -0.525534000 2.459589000 -3.105990000

H 0.123038000 3.250942000 -2.708154000

H -0.409799000 2.401442000 -4.191689000

H -1.558735000 2.737885000 -2.859017000

# *mer* elongation CF_3_-CH_3_ (PW91)

Mn 0.114382000 -0.140776000 0.224609000

O 1.951785000 0.609538000 0.123113000

O 0.684207000 -1.494873000 1.488147000

O -1.742067000 -0.829069000 0.288434000

O -0.394872000 1.159666000 1.858175000

O -0.417376000 1.163526000 -1.077149000

O 0.583149000 -1.541515000 -1.364912000

C 2.976980000 0.094684000 0.686360000

C 3.027634000 -1.022997000 1.510132000

C 1.879696000 -1.759050000 1.873854000

C -2.744682000 -0.224912000 0.823557000

C -2.751178000 0.843135000 1.703085000

C -1.571732000 1.472925000 2.206747000

C -0.340357000 1.107664000 -2.368670000

C 0.083911000 0.007965000 -3.125201000

C 0.507016000 -1.216764000 -2.581010000

H 3.988984000 -1.337071000 1.902984000

H -3.713697000 1.205636000 2.052017000

H 0.086086000 0.114458000 -4.205193000

C 0.956054000 -2.344091000 -3.555382000

F 0.863772000 -1.990482000 -4.872068000

F 2.255094000 -2.682090000 -3.312474000

F 0.194783000 -3.457248000 -3.373692000

C 4.284062000 0.870734000 0.377084000

F 4.208509000 2.138483000 0.868705000

F 5.385382000 0.279688000 0.924444000

F 4.481141000 0.952483000 -0.966295000

C 2.002529000 -2.954472000 2.775950000

H 1.322681000 -2.837313000 3.629099000

H 1.673375000 -3.845366000 2.224302000

H 3.025524000 -3.102649000 3.131323000

C -0.757501000 2.379549000 -3.054703000

H -0.102907000 3.196365000 -2.722427000

H -0.711122000 2.294133000 -4.143652000

H -1.778955000 2.639867000 -2.747461000

C -1.717711000 2.556301000 3.247966000

H -2.718256000 3.002158000 3.253056000

H -1.531327000 2.118097000 4.239175000

H -0.956950000 3.327792000 3.083311000

C -4.096793000 -0.830415000 0.372713000

F -4.251560000 -0.687279000 -0.976788000

F -4.153035000 -2.159431000 0.658523000

F -5.171894000 -0.235329000 0.970219000

# *mer* elongation CH_3_-CH_3_ (PW91)

Mn 0.322412000 -0.012640000 0.102784000

O 2.202095000 0.611228000 0.140174000

O 0.715026000 -1.377829000 1.429623000

O -1.531302000 -0.696049000 0.021973000

O -0.308203000 1.324431000 1.668060000

O -0.067119000 1.356086000 -1.199568000

O 0.742105000 -1.392474000 -1.503320000

C 3.134209000 0.098191000 0.846671000

C 3.041085000 -0.948683000 1.756491000

C 1.832081000 -1.630013000 2.008920000

C -2.561687000 -0.210093000 0.621828000

C -2.636285000 0.832247000 1.525584000

C -1.504751000 1.562817000 2.004882000

C -0.243946000 1.180157000 -2.468764000

C -0.093270000 0.034230000 -3.219927000

C 0.382939000 -1.214963000 -2.700997000

H 3.932954000 -1.255734000 2.292696000

H -3.616892000 1.102999000 1.904674000

H -0.325367000 0.091205000 -4.279323000

C 4.515912000 0.749806000 0.587921000

F 4.440590000 2.100812000 0.680719000

F 5.465960000 0.322958000 1.470931000

F 4.947450000 0.432941000 -0.666188000

C 1.785438000 -2.734994000 3.028906000

H 1.167805000 -2.408716000 3.877014000

H 1.293426000 -3.613917000 2.592833000

H 2.781213000 -3.003197000 3.392198000

C -1.741485000 2.705404000 2.960602000

H -2.672162000 2.590521000 3.527058000

H -0.887772000 2.802953000 3.638786000

H -1.810308000 3.635972000 2.377220000

C -3.857919000 -0.967568000 0.241315000

F -4.017079000 -1.005542000 -1.110986000

F -3.801542000 -2.256806000 0.687370000

F -4.980765000 -0.401168000 0.772393000

C 0.453770000 -2.390580000 -3.641864000

H 0.877399000 -2.104317000 -4.611931000

H 1.042284000 -3.193609000 -3.190368000

H -0.565764000 -2.759677000 -3.826415000

C -0.666160000 2.493274000 -3.168742000

F 0.338162000 3.411818000 -3.098326000

F -0.966194000 2.314401000 -4.487961000

F -1.763723000 3.031538000 -2.566619000
